# Factors associated with lung cancer among firefighters: a systematic literature review

**DOI:** 10.1186/s12889-025-21432-0

**Published:** 2025-01-22

**Authors:** Augustine W. Kang, Natalie S. Lui

**Affiliations:** 1 Department of Cardiothoracic Surgery, Stanford University School of Medicine, Stanford, CA USA; 2 Falk Cardiovascular Research Center, Stanford University School of Medicine, 300 Pasteur Drive, Stanford, CA 94305 USA

**Keywords:** Lung cancer, Firefighters, Occupational hazards, *Bacillus subtilis*

## Abstract

This short review addresses the pressing issue of lung cancer among firefighters, a population facing unique occupational hazards such as smoke inhalation and asbestos exposure. With lung cancer being a leading global cause of death, the study emphasizes the disproportionate burden on firefighters. Notably, wildfire smoke, containing carcinogenic elements, poses a rising significant threat to firefighters’ respiratory health. Despite evidence linking firefighting to increased lung cancer risk, few studies have explored the underlying mechanisms. This study conducts a comprehensive review employing PRISMA guidelines to synthesize existing literature from 1972 to 2022. We discuss the association between age, race, and time spent fighting fires with lung cancer development. Our review also underscores the scarcity of studies investigating specific carcinogens and their role in firefighters’ lung cancer risk, emphasizing the need for more targeted research. The study advocates for improved methodology, suggesting the use of individual-level exposure metrics like “fire-hours” to enhance causal inference. Despite limitations in current literature, the findings stress the urgency of understanding the intricacies of lung cancer development among firefighters and call for further research to inform preventive measures and potential screening protocols.

**Supplementary Information:**

The online version contains supplementary material available at 10.1186/s12889-025-21432-0.

## Introduction

Lung cancer is among the leading causes of death globally [1], with approximately 130,000 deaths from lung cancer just within the United States in 2022 [2]. The annual burden of disease is higher than that of any other cancer, and its average 5-year survival rate is the lowest among all types of cancers [3]. An important facet of lung cancer disease burden is the effect of premature mortality– the National Cancer Institute estimates that in the year 2009 alone, more than 2 million person-years of life were lost to lung cancer in the United States, which was significantly higher than any other type of cancer.

Firefighters encounter unique occupational hazards that increase the risk of lung cancer (e.g., smoke inhalation, asbestos exposure) that are known to be carcinogenic [4]. A study performed by the National Institute for Occupational Safety and Health among 30,000 firefighters (of approximately 1.2 million firefighters nationwide) reported a 9% increase in all-cancer diagnoses and a 14% increase in all cancer-related mortality relative to the general population [5, 6]. One of the main occupational hazards that firefighters face is exposure to smoke from various types of fires, including structure fires, vehicle fires, and wildland fires. Smoke from these sources contains a complex mixture of carcinogenic chemicals, such as benzene, formaldehyde, and polycyclic aromatic hydrocarbons (PAHs), which pose significant risks to respiratory and overall health. Notably, the increasing frequency and intensity of wildfires have heightened concerns about prolonged exposure to wildfire smoke among firefighters [7, 8]. This is concerning in light of a recent meta-analysis reporting an association between lung cancer risk and exposure to PM_2.5_ [9] a cohort study also reported increased risk of lung cancer (and cardiovascular mortality) due to exposure to PM_2.5_ [10]. The International Agency for Research on Cancer (IARC) recently classified occupational exposure as a firefighter as a Group 1 carcinogen, causally linked to mesothelioma and bladder cancer, though the evidence for lung cancer remains inconclusive with further research needed [11]. While some evidence has identified an association between firefighting and the development of lung cancer [12], no studies have examined the mechanisms underlying lung cancer development among firefighters.

To our knowledge, there has been no focused review of factors associated with lung cancer among firefighters. Hence, the goal of this study is to provide a narrative review of the published literature.

## Methods

This review was informed by the Preferred Reporting Items for Systematic Reviews (PRISMA) guidelines. A comprehensive literature search was conducted utilizing the PubMed (Medline) database. All searches were conducted in English and relevant articles published between 1972 and 2022 were obtained through the electronic database search. Search strategies, developed in consultation with a medical librarian, were: (“Lung Neoplasms“[mesh] OR (((lung*[tiab] OR lung*[tt] OR lung*[ot]) OR (bronch*[tiab] OR bronch*[tt] OR bronch*[ot]) OR (pulmon*[tiab] OR pulmon*[tt] OR pulmon*[ot])) AND ((cancer*[tiab] OR cancer*[tt] OR cancer*[ot]) OR (neopla*[tiab] OR neopla*[tt] OR neopla*[ot]) OR (tumor*[tiab] OR tumor*[tt] OR tumor*[ot]) OR (tumour*[tiab] OR tumour*[tt] OR tumour*[ot]) OR (carcinoma*[tiab] OR carcinoma*[tt] OR carcinoma*[ot]) OR (adenocarcinoma*[tiab] OR adenocarcinoma*[tt] OR adenocarcinoma*[ot]) OR (“small cell“[tiab] OR “small cell“[tt] OR “small cell“[ot]) OR (“squamous“[tiab] OR “squamous“[tt] OR “squamous“[ot]))) OR ((“NSLC“[tiab] OR “NSLC“[tt] OR “NSLC“[ot]) OR (“NSCLC“[tiab] OR “NSCLC“[tt] OR “NSCLC“[ot]) OR (“SLC“[tiab] OR “SLC“[tt] OR “SLC“[ot]) OR (“SCLC“[tiab] OR “SCLC“[tt] OR “SCLC“[ot]))) AND (“Firefighters“[all] OR firefight*[all] OR “firefighters”[mesh] OR “fire fight*”[all] OR fireman[all] OR firewom*[all] OR firemen[all] OR “fire person*”[all] OR “fireworker*”[all] OR “fire worker*”[all]).

### Abstract and full-text screening

Research studies that alluded to lung cancer among firefighters were selected for further review. Subsequently, the abstracts from the papers that were acquired by the searches were reviewed. If no abstract was available, information was extrapolated from the title of the paper. Following the abstract screening process, an inclusion criterion was used to assess each paper via a full-text screening process. Studies that met the following inclusion criteria were included: (a) study population included firefighters, (b) examined lung cancer as either exposure or outcome (c) publication date between 1972 and 2022, (d) published in the English language, (e) examined factors associated with lung cancer in their analysis. Studies were included if they specifically examined lung cancer as either the primary exposure or outcome of interest. Broader studies analyzing multiple cancers were excluded unless lung cancer was a dedicated focus of the analysis.

**Fig. 1:**
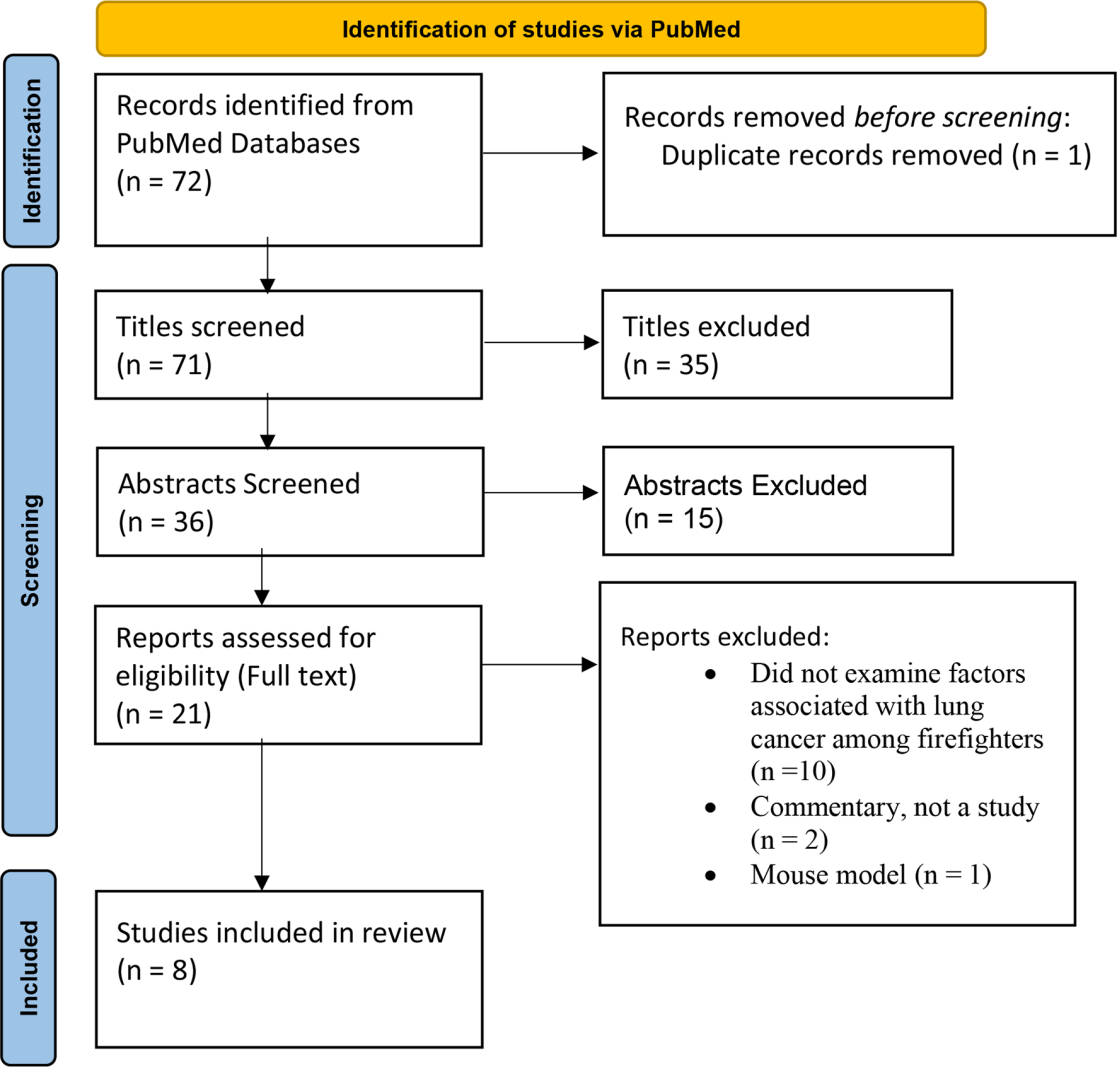
PRISMA diagram

Of the full-text articles assessed for eligibility, it was identified that a total of 8 studies (Fig. 1) met the inclusion criteria.

### Analysis and synthesis

Data were extracted from the included articles in detail, and the data were subsequently entered into Table 1, which includes the following data: Type of study number of participants, rating quality (per Oxford Center for Evidence-Based Medicine), and a summary of the study results. A synthesis of the studies’ findings in the context of explaining factors impacting firefighters’ lung health is reflected in the results section.

## Results

Table 1 presents the included studies and their main findings. Overall, there were eight studies, of which seven were retrospective cohort studies, and one was a pooled-analysis study. Most studies included a North American sample.

**Table 1:** Study results

#	Lead Author	Title	Type of study	Number of participants	Rating quality^1^	Main findings
1	Heyer, F., et al. [13]	Cohort mortality study of Seattle fire fighters: 1945-1983	Retrospective cohort study	2289	3	Increased standardized mortality ratio for lung cancer with time since first exposure (authors created 3-time groups: <15 years, 15–29 years, and 30 + years; SMR for 15–29 years = 55 (95% CI 18–127); SMR for 30 + years = 121 (95% CI 78–180).
2	Demers, P., et al. [14]	Mortality among firefighters from three northwestern United States cities	Retrospective cohort study of male firefighters	4401	3	The authors used years of service as a surrogate to smoke exposure. There were 95 deaths due to lung cancer with the standardized mortality ratio being 0.96 (95% CI 0.77–1.17). Years of service was not associated with lung cancer risk. Compared to the general population (White males), the authors found no difference in prevalence.
3	Guidotti, T. L.[15]	Mortality of urban firefighters in Alberta, 1927–1987	Retrospective cohort study of firefighters	3328	3	The authors examine only death due to lung cancer, but not total incidence of lung cancer. There were 27 cases of mortality due to lung cancer. Analysis by latency period (years of service) showed a peak risk at, or over 50 years after entry into the firefighting workforce. The authors further used an “exposure opportunity index” that suggested increased risk in less heavily exposed firefighters until 35 + years in service.
4	Ma, F., et al. [16]	Race-specific cancer mortality in US firefighters: 1984–1993	Retrospective cohort study of firefighters (deaths-only)	6607	3	Among 1883 cancer-related deaths, White firefighters (*n* = 1817) had increased risk of lung cancer (MOR = 1.1, 95% CI 1.0-1.2) relative to black firefighters (*n* = 66).
5	Pukkala, E. et al. [17]	Cancer incidence among firefighters: 45 years of follow-up in five Nordic countries	Retrospective cohort study of firefighters	16,422	3	Increased risk of adenocarcinoma (SIR = 1.29, 95% CI 1.02–1.60) among firefighters relative to the general population, but not squamous and small-cell carcinoma. Age was found to be a significant predictor– specifically, among firefighters over the age of 70 years, overall lung cancer prevalence was higher.
6	Daniels, R. et al. [18]	Exposure–response relationships for select cancer and non-cancer health outcomes in a cohort of US firefighters from San Francisco, Chicago and Philadelphia (1950–2009).^α^	Retrospective cohort study of firefighters	19,309	3	The authors found significant positive associations between a composite variable “fire-hours” and lung cancer mortality (1.39 95% CI: 1.12–1.73) and incidence (1.39 95% CI 1.10–1.74).
7	Bigert, C., et al. [19]	Lung cancer among firefighters: smoking-adjusted risk estimates in a pooled analysis of case-control studies	Pooled analysis (with meta-analysis) of case-control studies	32,301(14748 cases, 17543 controls); 86 participants identified to be firefighters.	1*	The main objective of the study was to explore lung cancer risk among firefighters, adjusting for tobacco use. The study population spanned across Europe, Canada, New Zealand, and China. The authors found no increased lung cancer (or by its subtypes) incidence before or after smoking adjustment.
8	Baris, D., et al. [20]	Cohort Mortality Study of Philadelphia Firefighters	Retrospective cohort study of firefighters	7,789	3	The authors reported a standardized mortality ratio of 1.13, 95% CI 0.97–1.32) which was not significant, “though elevated”.

^1^See Appendix A. *While the study design was a meta-analysis, this study did not focus only on the firefighter population. SMR = Standardized Mortality Ratio. ^α^ The authors published an update in 2020 featuring a 7-year extension of results, reporting that lung cancer SMR was 1.08 (95% CI 1.02–1.15) [21].

## Discussion

The association between firefighters and cancer (in particular, lung cancer) is of great interest because it is widely known that firefighters have especially frequent exposure to a variety to carcinogens in their work. Overall, 72 studies were identified from our search terms, and eight were eventually included for a narrative synthesis. While many studies have attempted to examine the prevalence and/or incidence of lung cancer among firefighters, our review showed that exceedingly few studies have attempted to identify the mechanisms and factors associated with lung cancer among firefighters. The few studies that exist may partially explain the IARC’s findings that the link between firefighting and lung cancer is inconclusive. The large, multi-year cohort studies included in our review offer relatively robust result inferences that compel our conclusions. Studies often utilized the infrastructure of existing health systems (e.g., Guidotti, 1993) that featured low attrition and high follow-up rates, which can improve the reliability of the results.

Most of the included studies (four of seven studies comparing lung cancer prevalence or incidence among firefighters) reported a higher prevalence of lung cancer among firefighters compared to the general population [13, 15, 17, 18]. Age was found to be associated with lung cancer incidence or mortality [13, 15, 17], consistent with other forms of cancer where advancing age is among the most salient risk factors for cancer incidence. One study reported that white race was associated with a slight but significantly increased risk of lung cancer relative to black firefighters [16]. However, there were significant limitations for the generalizability of the study findings due in part to a severely unequal sample size (i.e., 1817 White firefighters vs. 66 Black firefighters). Lastly, “fire-hours” (defined as an estimate of the actual duration of time spent fighting fires) was found to be associated with both lung cancer incidence and mortality.

Most cohort studies in our review used some form of surrogate measure to quantify occupational measure, most common of which is years in service. For example, Heyer and colleagues created time-based groups in their study population (< 15 years, 15–29 years, and 30 + years since first exposure) and compared lung cancer mortality between groups. However, it is unclear if “time since first exposure” is a form of cumulative occupational exposure, or if it refers to time spent in the present fire department (furthermore, it is also unclear if occupational attrition was accounted for). To quantify exposure, most of the included studies used employment records to estimate the duration and intensity for each firefighter, accounting for the duration and type of work performed.

Follow-up studies with first responder 9/11 firefighters identified an increased incidence of lung cancer [22]. While a recent epidemiological literature review on the risk of cancer among firefighters reported a moderate association between lung cancer and firefighting, it was unclear how firefighting was associated with poor health outcomes [23]. The lack of specificity in occupational exposure was addressed to a great extent by Daniels and colleagues in their cohort study of US firefighters from San Francisco, Chicago, and Philadelphia. This study filled gaps in literature by accounting for firefighter exposures in cancer risk assessment; Specifically, the authors were able to derive an estimate of the total number of time spent at fires for each firefighter (“fire hours”), hence reflecting a more accurate, individual-level exposure to occupational hazards as opposed to using time in job as a surrogate measure. In doing so, the authors were able to demonstrate (for the first time) exposure-response associations in lung cancer development [18]. Future studies attempting to establish causality between exposure and outcomes among firefighters may consider using a similar approach.

The only meta-analysis included in our review (Bigert et al., 2016) controlled for smoking and found no increased risk of lung cancer overall (or by its subtypes). However, a major limitation (acknowledged by the authors) was the small sample size of firefighters (*n* = 86 with lung cancer), which severely limits the power to detect excess risks of lung cancer. There was also large heterogeneity in the characteristics of the included firefighters, further increasing variance and the generalizability of its results. While high prevalence of smoking among firefighters has been a concern for increased lung cancer risk, a recent study reported declining trends in smoking among firefighters [24]. Regardless, firefighters are exposed to a wide variety of carcinogenic products (including arsenic, asbestos, benzene, cadmium, and silica) in their line of work, but no studies have examined these specific factors and their association with lung cancer in firefighters. Hence, it remains unclear how occupational hazards experienced in the line of duty affects lung cancer risk.

While the current literature identifies an association between firefighting and lung cancer, the specific biological mechanisms underlying this risk remain unclear and warrant further investigation. Fire smoke—whether from structural, vehicle, or wildland fires—contains a mixture of known carcinogens, including polycyclic aromatic hydrocarbons (PAHs), benzene, formaldehyde, and fine particulate matter (PM2.5). PAHs and benzene, in particular, are well-documented carcinogens that can be inhaled during fire suppression activities and contribute to DNA damage, oxidative stress, and subsequent carcinogenesis [11]. Research using individual-level exposure metrics, such as ‘fire-hours’ or specific exposure assessments for PAHs and PM2.5, is essential to delineate the relationship between cumulative exposures and lung cancer development more precisely. Further mechanistic studies are also needed to explore how occupational exposures interact with other risk factors, such as smoking or genetic predispositions, to influence carcinogenesis among firefighters.

In summary, our review findings suggests that age, race, and time spent fighting fire are associated with the development of lung cancer among firefighters and highlights the presence of a gap in the literature in identifying mechanisms of lung cancer among firefighters. Our review complements the IARC conclusions by synthesizing factors like age, race, and occupational exposure proxies, while identifying gaps (e.g., lack of mechanistic studies and accurate exposure measures) [11]. However, many studies included in our review have significant limitations that restrict the generalizability of their findings. One limitation of relying on death certificates to determine cause of death is its relative inaccuracy (compared to a pathological autopsy), though these limitations may be attenuated to some extent by the studies’ robust sample sizes. In addition, future studies attempting to delineate factors underlying lung cancer in firefighters should consider examining individual-level exposure (e.g., “fire-hours” instead of surrogate measures (e.g., time in job) to establish causality more accurately, and to examine specific carcinogens experienced by firefighters to better understanding their unique occupational risks. We also acknowledge that our inclusion criteria may have excluded studies reporting lung cancer risk as part of a broader analysis of multiple cancers. For example, a study by Tsai et al. (2015) presented valuable data on lung cancer among firefighters but was not included due to its broader focus [25]. This exclusion criterion may potentially introduce a selection bias toward studies emphasizing positive associations, In addition, while lung cancer screening using annual low dose computed tomography has been recommended for people who smoke, further research is needed to determine if firefighters might also benefit from lung cancer screening. Notably, the National Firefighter Registry (NFR) for Cancer, launched by NIOSH in 2023, represents a significant step forward in addressing these gaps. The NFR contains comprehensive health and occupational exposure data from U.S. firefighters, which may allow for a better understanding of cancer risk [26].

A major limitation of the current literature is the lack of information on the use of personal protective equipment (PPE), particularly respiratory protection, among firefighters. Proper and consistent use of PPE, such as self-contained breathing apparatuses (SCBAs), could substantially mitigate exposure to carcinogenic chemicals present in smoke. However, PPE usage varies across different fire scenarios, departments, and historical time periods, which may contribute to heterogeneity in observed lung cancer risks. Additionally, the included studies did not consistently differentiate between types of fires (e.g., structure fires, vehicle fires, or wildland fires), each of which involves unique chemical exposures. The absence of detailed exposure classification prevents a more refined analysis of how specific types of fires contribute to lung cancer risk.

## Electronic supplementary material

Below is the link to the electronic supplementary material.

Supplementary Material 1

## Data Availability

The authors confirm that the data supporting the findings of this study are available within the article and its supplementary materials.

## Abbreviations

PRISMA: Preferred Reporting Items for Systematic Reviews

## References

[1] Adjei AA. Lung cancer worldwide. J Thorac Oncol. 2019;14(6):956. 31122558 10.1016/j.jtho.2019.04.001

[2] American Cancer Society. Key Statistics for Lung Cancer. American Cancer Society. Accessed Jan 13 2023, https://www.cancer.org/research/cancer-facts-statistics/all-cancer-facts-figures/cancer-facts-figures-2022.html

[3] Wender R, Fontham ET, Barrera E Jr, et al. American Cancer Society lung cancer screening guidelines. Cancer J Clin. 2013;63(2):106–17. 10.3322/caac.21172 PMC3632634 23315954

[4] Fritschi L, Glass DC. Firefighters and cancer: where are we and where to now? BMJ Publishing Group Ltd; 2014. pp. 525–6. 10.1136/oemed-2014-102230 24996680

[5] Daniels RD, Kubale TL, Yiin JH, et al. Mortality and cancer incidence in a pooled cohort of US firefighters from San Francisco, Chicago and Philadelphia (1950–2009). Occup Environ Med. 2014;71(6):388–97. 24142974 10.1136/oemed-2013-101662 PMC4499779

[6] United States Fire Administration. National Fire Department Registry Quick Facts. Accessed Jan 10 2023, https://apps.usfa.fema.gov/registry/summary

[7] Adetona O, Reinhardt TE, Domitrovich J, et al. Review of the health effects of wildland fire smoke on wildland firefighters and the public. Inhalation Toxicol. 2016;28(3):95–139. 10.3109/08958378.2016.1145771 26915822

[8] Navarro KM, Kleinman MT, Mackay CE, et al. Wildland firefighter smoke exposure and risk of lung cancer and cardiovascular disease mortality. Environ Res. 2019;173:462–8. 30981117 10.1016/j.envres.2019.03.060

[9] Hamra GB, Guha N, Cohen A et al. Outdoor particulate matter exposure and lung cancer: a systematic review and meta-analysis. Environ Health Perspect. 2014. 10.1289/ehp/1408092 PMC4154221 24911630

[10] Pun VC, Kazemiparkouhi F, Manjourides J, Suh HH. Long-term PM2. 5 exposure and respiratory, cancer, and cardiovascular mortality in older US adults. Am J Epidemiol. 2017;186(8):961–9. 28541385 10.1093/aje/kwx166 PMC6915823

[11] International Agency for Research on Cancer. 132: Occupational exposure as a firefighter. IARC Monogr Identif Carcinog Hazards Hum. 2023. 37963216

[12] Soteriades ES, Kim J, Christophi CA, Kales SN. Cancer Incidence and Mortality in firefighters: a state-of-the-art review and Meta-analysis. Asian Pac J Cancer Prev Nov. 2019;1(11):3221–31. 10.31557/APJCP.2019.20.11.3221. 10.31557/APJCP.2019.20.11.3221 PMC7063017 31759344

[13] Heyer N, Weiss NS, Demers P, Rosenstock L. Cohort mortality study of Seattle fire fighters: 1945–1983. Am J Ind Med. 1990;17(4):493–504. 10.1002/ajim.4700170407. 2327416 10.1002/ajim.4700170407

[14] Demers PA, Heyer NJ, Rosenstock L. Mortality among firefighters from three northwestern United States cities. Occup Environ Med. 1992;49(9):664–70. 10.1136/oem.49.9.664 PMC1039313 1390274

[15] Guidotti TL. Mortality of urban firefighters in Alberta, 1927–1987. Am J Ind Med. 1993;23(6):921–40. 8328477 10.1002/ajim.4700230608

[16] Ma F, Lee DJ, Fleming LE, Dosemeci M. Race-specific cancer mortality in US firefighters: 1984–1993. J Occup Environ Med. 1998:1134–8. 10.1097/00043764-199812000-00014 9871891

[17] Pukkala E, Martinsen JI, Weiderpass E, et al. Cancer incidence among firefighters: 45 years of follow-up in five nordic countries. Occup Environ Med. 2014;71(6):398–404. 24510539 10.1136/oemed-2013-101803

[18] Daniels RD, Bertke S, Dahm MM, et al. Exposure–response relationships for select cancer and non-cancer health outcomes in a cohort of US firefighters from San Francisco, Chicago and Philadelphia (1950–2009). Occup Environ Med. 2015;72(10):699–706. 25673342 10.1136/oemed-2014-102671 PMC4558385

[19] Bigert C, Gustavsson P, Straif K, et al. Lung cancer among firefighters: smoking-adjusted risk estimates in a pooled analysis of case-control studies. J Occup Environ Med. 2016;58(11):1137. 27820764 10.1097/JOM.0000000000000878 PMC7254920

[20] Baris D, Garrity TJ, Telles JL, Heineman EF, Olshan A, Zahm SH. Cohort mortality study of Philadelphia firefighters. Am J Ind Med. 2001;39(5):463–76. 11333408 10.1002/ajim.1040

[21] Pinkerton L, Bertke SJ, Yiin J, et al. Mortality in a cohort of US firefighters from San Francisco, Chicago and Philadelphia: an update. Occup Environ Med. 2020;77(2):84–93. 31896615 10.1136/oemed-2019-105962 PMC10165610

[22] Printz C. New research finds lung cancer screening guidelines are insufficient for firefighters. Cancer. 2020;126(4):692–3. 31995245 10.1002/cncr.32721

[23] Brantom PG, Brown I, Baril M, McNamee R. Epidemiol Literature Rev Risk Cancer among Firefighters. 2018.

[24] Phan L, McNeel TS, Jewett B, Moose K, Choi K. Trends of cigarette smoking and smokeless tobacco use among US firefighters and law enforcement personnel, 1992–2019. Am J Ind Med. 2022;65(1):72–7. 34766643 10.1002/ajim.23311 PMC8678355

[25] Tsai RJ, Luckhaupt SE, Schumacher P, Cress RD, Deapen DM, Calvert GM. Risk of cancer among firefighters in California, 1988–2007. Am J Ind Med. 2015;58(7):715–29. 25943908 10.1002/ajim.22466 PMC4527530

[26] Fent KW, Siegel M, Mayer A, Wilkinson A. National Firefighter Registry (NFR) protocol. 2022. 10.1002/ajim.70112 PMC13393521 42449181

