# Supplementary material for: Factors associated with lung cancer among firefighters: a systematic literature review
Source: BMC Public Health. 2025 Jan 22;25:281. doi: 10.1186/s12889-025-21432-0 (PMC11755900; doi:10.1186/s12889-025-21432-0)
Supplement: Supplementary file 1 — Supplementary Material 1 [file 12889_2025_21432_MOESM1_ESM.docx]

Appendix A. Quality Rating Scheme

|  | Quality Rating Scheme |
| --- | --- |
| 1 | Properly powered and conducted randomized clinical trial; systematic review with meta-analysis |
| 2 | Well-designed controlled trial without randomization; prospective comparative cohort trial |
| 3 | Case-control studies; retrospective cohort study |
| 4 | Case series with or without intervention; cross-sectional study |
| 5 | Opinion of respected authorities; case reports |
